# Mortality Outcomes and Angiotensin-Converting Enzyme Inhibitor Use in Patients With Idiopathic Pulmonary Fibrosis

**DOI:** 10.1016/j.chest.2025.07.4077

**Published:** 2025-08-14

**Authors:** Burcu Ozaltin, Robert Chapman, Tine Follet, Marie Vermant, Muhammad Qummer Ul Arfeen, Natalie Fitzpatick, Harry Hemingway, Wim Wuyts, Kenan Direk, Joseph Jacob

**Affiliations:** a Satsuma Lab, Hawkes Institute, University College London, London, England; b UCL Respiratory, University College London, London, England; c UCL Institute of Health Informatics, University College London, London, England; d Department of Respiratory Diseases, Unit for Interstitial Lung Diseases, University Hospitals Leuven, Leuven, Belgium; e Imperial Clinical Trials Unit, School of Public Health, Imperial College London, London, England

**Keywords:** ACE inhibitor, antifibrotic, COPD, drug repurposing, fibrosis, idiopathic pulmonary fibrosis, mortality

## Abstract

**Background:**

Angiotensin-converting enzyme (ACE) inhibitors are widely used antihypertensive agents with proven cardioprotective effects. Previous mechanistic and clinical studies have suggested that ACE inhibitor therapy may slow disease progression and reduce mortality in idiopathic pulmonary fibrosis (IPF).

**Research Question:**

Does ACE inhibitor use associate with reduced all-cause mortality in a real-world population of patients with IPF, and if so, is the association also present in patients with COPD?

**Study Design and Methods:**

A retrospective analysis was conducted by using electronic health records from the Clinical Practice Research Datalink GP Online Database (2019), linked with Hospital Episode Statistics Admitted Patient Care and Office for National Statistics death registration data. Patients with IPF and COPD were stratified based on ACE inhibitor use (defined as ≥ 3 prescriptions within the 5 years preceding diagnosis) and matched by age, sex, and smoking history using propensity score matching. Multivariable Cox regression analyses were performed, adjusting for age, sex, BMI, smoking status, indices of multiple deprivation, diabetes mellitus, chronic kidney disease, and common cardiovascular comorbidities. In the IPF cohort, competing risk analysis was used to account for cause-specific mortality.

**Results:**

The study included 3,579 patients with IPF and matched COPD control participants (mean age, 74 years; 36% female). Among the IPF cohort, 1,328 (37%) were ACE inhibitor users, compared with 1,061 (30%) among patients with COPD. ACE inhibitor use was associated with improved survival, independent of coexisting comorbidities, in patients with IPF (hazard ratio, 0.82; 95% CI, 0.75-0.91; *P* ≤ .001), but a similar association was not found in patients with COPD (hazard ratio, 1.09; 95% CI, 0.96-1.23; *P* = .180).

**Interpretation:**

In this study, ACE inhibitor therapy was independently associated with reduced all-cause mortality in IPF but not in COPD. Prospective trials are warranted to confirm these findings in IPF populations.

## Graphical Abstract

**Figure:**
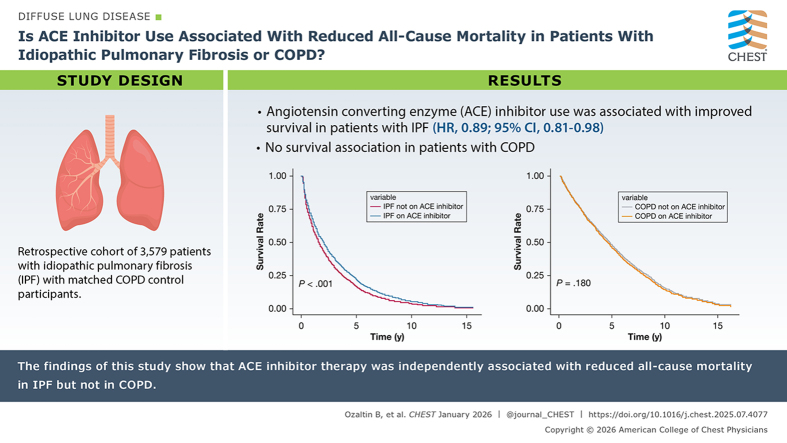

FOR EDITORIAL COMMENT, SEE PAGE 6

**Take-Home Points**

**Research Question:** Does angiotensin-converting enzyme (ACE) inhibitor therapy reduce all-cause mortality in patients with idiopathic pulmonary fibrosis (IPF), and is this association also seen in COPD?

**Results:** In a large, real-world cohort, ACE inhibitor use was associated with a statistically significant reduction in all-cause mortality in IPF but showed no survival benefit in COPD.

**Interpretation:** ACE inhibitor therapy may confer a disease-specific survival advantage in IPF, supporting the need for prospective trials to confirm its potential therapeutic role.

Idiopathic pulmonary fibrosis (IPF) is a progressive interstitial lung disease of unknown etiology.^1^ It typically presents in individuals aged > 50 years, predominantly affecting male individuals with a history of tobacco use.^1^, ^2^, ^3^ The disease course of IPF is highly variable and challenging to predict, with a median survival time of 3 to 5 years.^4^

Comorbidities are common in IPF and significantly affect mortality.^5^^,^^6^ Conditions such as arterial hypertension,^7^^,^^8^ diabetes mellitus,^8^ and other cardiovascular comorbidities are more prevalent in patients with IPF than in general smoking populations, contributing to a reduced quality of life.^8^, ^9^, ^10^, ^11^ Although no curative treatments exist for IPF, 2 antifibrotic therapies, pirfenidone and nintedanib, have shown efficacy in slowing disease progression, thereby improving outcomes.^12^ However, additional therapies to further reduce morbidity and mortality are urgently needed.

Angiotensin-converting enzyme (ACE) inhibitors are widely used to manage arterial hypertension and congestive heart failure,^13^^,^^14^ in which they have shown efficacy in attenuating cardiac remodeling.^15^^,^^16^ In the context of drug repurposing, there is growing interest in the broader pharmacologic effects of ACE inhibitors, particularly their role in modulating fibroproliferative processes across multiple organ systems.^17^, ^18^, ^19^, ^20^

The aim of the current study was to evaluate the association between ACE inhibitor use and mortality in patients with IPF identified by using secondary health care records.

## Study Design and Methods

### Data Source

We studied pseudonymized patient-level, electronic health care records from the Clinical Practice Research Data (CPRD) GP Online Database (January 2019), Hospital Episode Statistics Admitted Patient Care and Office for National Statistics death registration data, and small area level data based on patient postcode. A detailed description of this resource has been published previously.^21^

### Study Population

A cohort of patients with IPF diagnosed between January 1, 2002, and January 1, 2019, was established from the cohort of patients with multiple registrations using disease codes from the International Classification of Diseases, 10th Revision (ICD-10). The date of the first attendance during which an ICD-10 code mentioned IPF was taken as the date of diagnosis. Patients with IPF in which the date of the initial attendance with an ICD-10 code for IPF was on the date of death, as well as patients with IPF whose death date was < 60 days after having been diagnosed, were excluded. The study population comprised patients with IPF, with an age criterion restricting participants to those aged between 40 and 85 years (40 years ≤ age ≤ 85 years). This age range was selected to encompass the primary demographic group affected by IPF. Patients with IPF with a history of a rheumatologic disease (identified by review of ICD-10 codes) were excluded from the analyses.

Patients with COPD were 1:1 propensity matched with patients with IPF according to patient age, sex, and smoking duration, all at the time of the first COPD diagnosis. The cohort derivation process, including all inclusion and exclusion steps, is summarized in the Consolidated Standards of Reporting Trials-style diagram (e-Fig 1).

### Statistical Methods

Data are presented as means and medians (Q1-Q3) or as numbers of patients with percentages, as appropriate. Follow-up time was defined as the interval between the date of IPF or COPD diagnosis and the date of death or the last recorded follow-up. For the cohort analyses, the primary outcome measure was the mortality risk in patients using an ACE inhibitor (≥ 3 prescriptions within the 5 years prior to the IPF or COPD diagnosis date) compared with those not using an ACE inhibitor.

Time-to-death analyses were conducted by using univariable and multivariable Cox proportional hazards regression models to identify factors associated with mortality risk. In addition to estimating hazard ratios (HRs), adjusted survival curves were generated from the final multivariable Cox model. Covariates adjusted for in the models included patient age (at diagnosis of IPF/COPD), sex, smoking status (person who has never smoked, who formerly smoked, or who currently smokes), BMI, indices of multiple deprivation, diabetes mellitus, chronic kidney disease, pulmonary hypertension, and common cardiovascular comorbidities (hypertension and heart failure) at the time of IPF or COPD diagnosis. Comorbidities were identified through review of ICD-10 codes. The proportional hazards assumption was assessed by using Schoenfeld residuals, martingale residuals were used to check for nonlinearity, and deviance residuals were evaluated for the presence of influential observations.

A sensitivity analysis was performed in which the 60-day postdiagnosis survival restriction was removed to assess whether the observed association between ACE inhibitor use and mortality persisted when early deaths were included. This analysis used the same multivariable Cox modeling strategy applied to the broader IPF cohort. In addition, a competing risks analysis was performed by using the Fine and Gray subdistribution hazard model to assess the association between ACE inhibitor use and cause-specific mortality in IPF. Respiratory-related death was defined as the event of interest, while cardiovascular-related death and death from other causes were treated as explicit competing events. This approach allows for estimation of subdistribution HRs while accounting for the competing nature of different mortality causes. Cumulative incidence functions were also plotted to visually compare cause-specific mortality between ACE inhibitor users and nonusers.

Missing values for BMI at diagnosis of IPF/COPD (11%-16% missing) were imputed by using multiple imputation by chained equations, but no imputation or extrapolation was required for other clinical variables because there were no missing values. *P* values < .05 were considered statistically significant.

All statistical analyses were performed by using R versions 4.4.0/4.4.1 (R Foundation for Statistical Computing). Risk analyses were conducted by using the “survival” and “cmprsk” packages, while visualization of survival analysis results was facilitated by the “ggplot2,” “survminer,” “riskRegression,” and “forestplot” packages. The "mice" package was used for imputing missing BMI values.

### Ethics and Consent

The CPRD holds comprehensive ethics approval from the National Research Ethics Service Committee for conducting observational research using primary care data and preexisting data linkages. Because this study used routinely collected clinical data from the Cardiovascular Disease Research Using Linked Bespoke Studies and Electronic Health Records (CALIBER) study, informed consent from participants or their parents/guardians was not required. Ethical approval for the use of these data has already been established for observational research in CPRD.

This study was conducted in compliance with the ethical principles outlined in the 1964 Declaration of Helsinki and its subsequent amendments. Permission to access the data was granted by the Independent Scientific Advisory Committee at the Medicines and Healthcare products Regulatory Agency under Protocol No: 18_291, in accordance with Section 251 of the National Health Service Act 2006.

## Results

### Baseline Characteristics

A total of 3,579 patients with IPF were included in the cohort study and matched 1:1 with 3,579 patients with COPD for mortality comparison. Each of the 2 disease cohorts was then dichotomized according to ACE inhibitor use to form 2 groups within each cohort. A total of 1,328 patients with IPF used ACE inhibitors, whereas 1,061 patients with COPD used ACE inhibitors. The mean age across the 2 cohorts was 74 years, and 36% of the study participants were female. Smoking status and BMI were similar between ACE inhibitor users and nonusers with IPF and within the same 2 groups in patients with COPD. However, fewer patients with COPD were people who had never smoked, and there were fewer deaths in the COPD cohort compared with the IPF cohort (Table 1). Prevalence of comorbidities between ACE inhibitor user groups differed in both disease cohorts, with those using ACE inhibitors having significantly more comorbidities, particularly affecting the cardiovascular system (Table 2).

**Table 1:** Baseline Characteristics of the Study Population, Stratified According to Disease Cohort (IPF or COPD) and ACE Inhibitor Use

Demographic Characteristic	IPF (n = 3,579)		COPD (n = 3,579)	
	ACE Inhibitor Users (n = 1,328)	ACE Inhibitor Nonusers (n = 2,251)	ACE Inhibitor Users (n = 1,062)	ACE Inhibitor Nonusers (n = 2,517)
Age, y	77.10 (81.12-71.88)	75.80 (80.30-69.20)	76.95 (81.20-71.40)	76.10 (80.50-69.70)
Sex				
Male	903 (68)	1,403 (62)	707 (67)	1,599 (64)
Female	425 (32)	848 (38)	354 (33)	918 (36)
BMI				
Mean BMI, kg/m^2^	27.51	25.88	27.39	25.58
Not recorded	16%	24%	11%	18%
Smoking history				
Never smoked	265 (20)	467 (21)	65 (6)	169 (7)
Formerly smoked	785 (59)	1,215 (54)	572 (54)	1,114 (44)
Currently smokes	278 (21)	569 (25)	424 (40)	1,234 (49)
Status				
Alive	211 (16)	351 (16)	345 (33)	825 (33)
Dead (all causes)	1,117 (84)	1,900 (84)	717 (67)	1,692 (67)
Cause of death				
Respiratory death	596 (45)	1,074 (48)	233 (22)	658 (26)
Cardiac death	245 (18)	254 (11)	160 (15)	301 (12)
Region of England				
North	430 (32)	719 (32)	297 (28)	741 (29)
South	898 (68)	1,532 (68)	764 (72)	1,776 (71)
Overall follow-up, y	1.56 (3.33-0.63)	1.47 (3.27-0.57)	2.97 (5.62-1.31)	3.31 (6.01-1.42)
Survival time				
Follow-up time to death (all causes)	1.40 (3.10-0.60)	1.40 (3.00-0.50)	2.90 (5.70-1.20)	3.40 (6.00-1.40)
Follow-up time if alive	2.10 (4.85-1.00)	2.20 (4.30-0.80)	3.20 (5.50-1.40)	3.20 (6.10-1.40)

Data are presented as median (Q1-Q3) or No. (%) unless otherwise indicated. Percentages reflect the proportion within each ACE inhibitor use subgroup. ACE = angiotensin-converting enzyme; IPF = idiopathic pulmonary fibrosis.

**Table 2:** Prevalence of Comorbidities at the Time of Diagnosis, Stratified According to Disease Cohort (IPF or COPD) and ACE Inhibitor Use

Comorbidity	IPF on ACE Inhibitor (n = 1,328)	IPF With No ACE Inhibitor (n = 2,251)	COPD on ACE Inhibitor (n = 1,061)	COPD With No ACE Inhibitor (n = 2,517)
Hypertension	1,155 (87)	1,100 (49)	977 (92)	1,073 (43)
Cardiac disease	782 (59)	713 (32)	406 (38)	560 (22)
Heart failure	527 (40)	388 (17)	84 (8)	83 (3)
Diabetes	485 (37)	442 (20)	308 (29)	286 (11)
Chronic kidney disease	474 (36)	414 (18)	303 (29)	268 (11)
Pneumonia	468 (35)	776 (34)	205 (19)	464 (18)
Myocardial infarction	443 (33)	259 (12)	179 (17)	224 (9)
Neoplasm	372 (28)	637 (28)	286 (27)	634 (25)
Abdominal and peripheral	334 (25)	377 (17)	230 (22)	338 (13)
Cerebrovascular disease	141 (11)	162 (7)	123 (12)	196 (8)
Pulmonary hypertension	88 (7)	102 (5)	10 (1)	12 (< 1)
Lung cancer	59 (4)	102 (5)	29 (3)	90 (4)
Sleep apnea	53 (4)	61 (3)	25 (2)	33 (1)
Liver cirrhosis	17 (1)	41 (2)	11 (1)	37 (1)

Data are presented as counts and percentages within each ACE inhibitor use subgroup. Comorbidities were identified by using International Classification of Diseases, 10th Revision, codes recorded prior to or on the date of diagnosis. ACE = angiotensin-converting enzyme; IPF = idiopathic pulmonary fibrosis.

### ACE inhibitor Use and Survival in IPF

**Figure 1:**
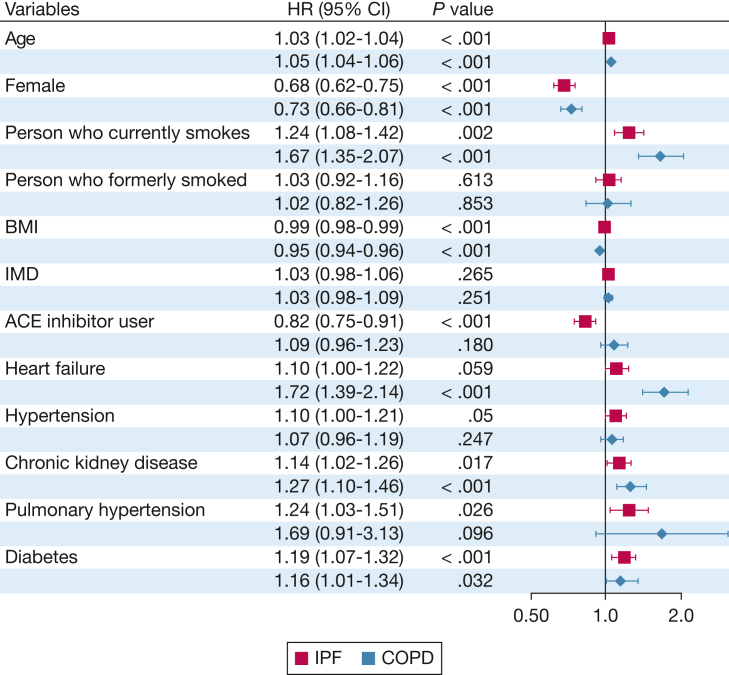
Forest plot displaying results from multivariable Cox proportional hazards regression models for all-cause mortality in patients with IPF and COPD. HRs are shown with corresponding 95% CIs and *P* values. Analyses were stratified according to disease group: red bars represent IPF, and blue bars represent COPD. Covariates included ACE inhibitor use and relevant demographic and clinical factors at diagnosis. ACE = angiotensin-converting enzyme; HR = hazard ratio; IMD = Index of Multiple Deprivation; IPF = idiopathic pulmonary fibrosis.

**Figure 2:**
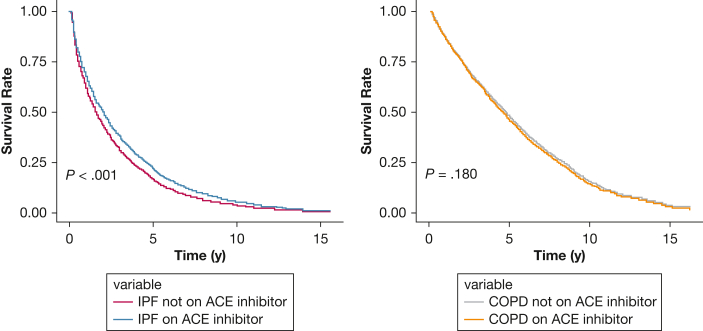
Adjusted survival curves comparing ACE inhibitor users and nonusers, derived from multivariable Cox proportional hazards regression models. Separate curves are shown for the IPF and COPD cohorts. Models were adjusted for age, sex, smoking status, BMI, deprivation index, and relevant comorbidities at diagnosis. ACE = angiotensin-converting enzyme; IPF = idiopathic pulmonary fibrosis.

In the IPF cohort, there were 1,117 (84%) deaths in the ACE inhibitor user group vs 1,900 (84%) deaths in the nonuser group. Kaplan-Meier curves showed no significant difference in unadjusted survival between ACE inhibitor users and nonusers (log-rank test, *P* = .72) (e-Fig 2). In univariable Cox regression analysis, ACE inhibitor use was not significantly associated with survival (HR, 0.99; 95% CI, 0.92-1.06; *P* = .72) (e-Table 1). On multivariable Cox regression analysis, ACE inhibitor use was independently associated with reduced mortality: HR, 0.82; 95% CI, 0.75-0.91; *P* < .001. In the same model, female sex also displayed a protective effect, whereas increasing age and current smoking status were significantly associated with increased mortality (Fig 1). Adjusted survival curves from the multivariable Cox model showed a higher survival probability for ACE inhibitor users over time (Fig 2).

To evaluate the robustness of the study findings, a sensitivity analysis was performed in which the 60-day postdiagnosis exclusion was removed. In this broader cohort, ACE inhibitor use remained significantly associated with reduced all-cause mortality (HR, 0.87; 95% CI, 0.80-0.94; *P* < .001) (e-Fig 3).

### Competing Risk Analysis in IPF

**Figure 3:**
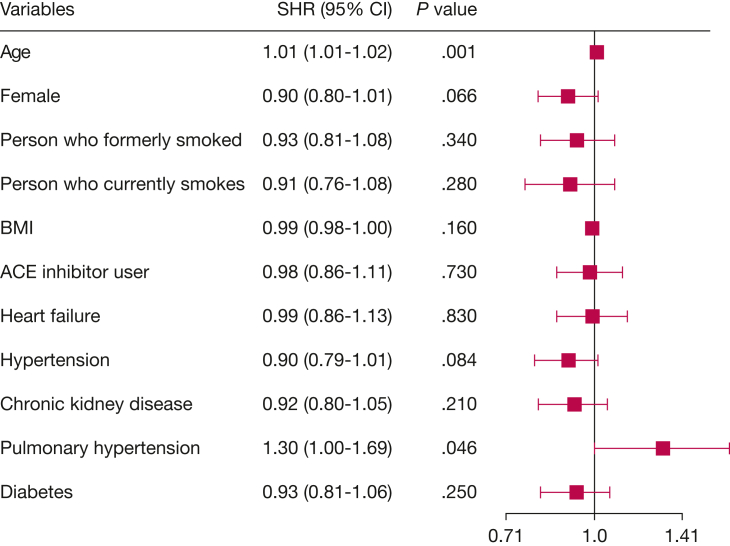
Forest plot displaying results from the competing risk regression model in the IPF cohort. The SHRs represent the risk of respiratory-related death, accounting for competing risks of cardiovascular and other causes of death. SHR values are shown with corresponding 95% CIs and *P* values. Event coding: 0 = alive (censored); 1 = respiratory death (event of interest); 2 = cardiovascular death; and 3 = other causes. ACE = angiotensin-converting enzyme; IPF = idiopathic pulmonary fibrosis; SHR = subdistribution hazard ratio.

**Figure 4:**
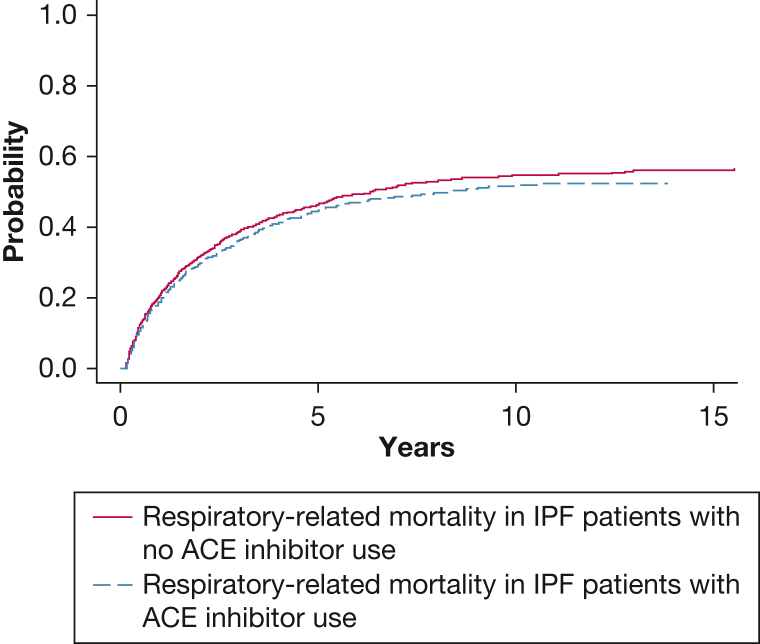
Cumulative incidence curves for respiratory-related mortality in the IPF cohort, estimated by using a competing risks model. The curves compare ACE inhibitor users and nonusers, with cardiovascular and other causes of death treated as competing events. This analysis corresponds to the multivariable subdistribution hazard model presented in Figure 3. ACE = angiotensin-converting enzyme; IPF = idiopathic pulmonary fibrosis.

The study of mortality in the IPF population was further examined by using a multivariable competing risk model. In patients using ACE inhibitors, 45% of deaths were respiratory related and 18% were cardiovascular related, compared with 48% and 11% of deaths in the ACE inhibitor nonusers, respectively (Table 1). However, in the analysis explicitly accounting for all competing causes of death, ACE inhibitor use was not significantly associated with a reduction in respiratory-related mortality: subdistribution HR, 0.98; 95% CI, 0.86-1.11; *P* = .730 (Fig 3). In the same model, female sex similarly exhibited a risk reduction effect, whereas advancing age increased the risk of respiratory-related death (Fig 2). Comorbid diagnoses of heart failure, hypertension, chronic kidney disease, and diabetes mellitus did not influence the risk of respiratory-related death in this model. Cumulative incidence functions for respiratory-related mortality by ACE inhibitor use are presented in Figure 4.

### Comparison vs Matched COPD Cohort

In the matched COPD cohort, there were 717 (20%) deaths in ACE inhibitor users and 1,692 (47%) deaths in nonusers. On multivariable analysis (using the same covariates as those in the IPF cohort analysis), ACE inhibitor use did not have a significant effect on mortality (HR, 1.09; 95% CI, 0.96-1.23; *P* = .180). The protective effect of female sex was again shown, with increasing BMI also exhibiting an association with reduced mortality in this cohort (Fig 1). Advancing age, current smoking, heart failure, chronic kidney disease, and diabetes mellitus were all associated with increased mortality in patients with COPD. Adjusted survival curves from the multivariable Cox model for the COPD cohort showed no appreciable difference in survival between ACE inhibitor users and nonusers (Fig 2).

## Discussion

The current cohort study, to our knowledge, is the largest to assess outcomes in patients with IPF using ACE inhibitors. The results underscore the complex relationship between angiotensin-modulating drugs and IPF pathogenesis, suggesting a potential role for ACE inhibitors as disease-modifying agents in IPF therapy. These findings indicate that ACE inhibitor therapy, initiated within 5 years prior to IPF diagnosis, was independently associated with reduced all-cause mortality on multivariable modeling, highlighting the important role of confounding factors in real-world clinical data and the potential for these to mask true therapeutic effects. In competing risk analysis, ACE inhibitor use was not associated with a specific reduction in respiratory-related mortality, suggesting instead a broader multisystem effect that reduces overall mortality risk. We found no evidence of a mortality benefit associated with ACE inhibitor use in the matched COPD cohort, indicating that the survival advantage observed in IPF is unlikely to be explained by general cardiovascular effects alone. This distinction suggests that the survival benefit observed in IPF may be uniquely linked to IPF-specific mechanisms, indicating a potential disease-specific effect of ACE inhibitors.

Previous studies evaluating ACE inhibitor therapy in IPF have yielded mixed results. A post hoc analysis of data from the Clinical Studies Assessing Pirfenidone in IPF: Research of Efficacy and Safety Outcomes (CAPACITY) and Efficacy and Safety of Pirfenidone in Patients With Idiopathic Pulmonary Fibrosis (ASCEND) trials (624 patients) found an association between ACE inhibitor use and slower IPF progression, as well as a trend toward reduced IPF-related mortality, although the latter was not statistically significant.^22^ In addition, a prospective study of patients with oxygen-dependent pulmonary fibrosis (462 patients) reported a reduction in mortality among those using angiotensin modulators.^23^ Conversely, other retrospective analyses of IPF cohorts reported no protective effect from ACE inhibitor treatment, although these studies included relatively small numbers of ACE inhibitor users.^24^^,^^25^

Studies in both animal and human models show that angiotensin II levels are elevated in IPF lungs, while ACE2 (an enzyme critical for angiotensin II degradation) expression is reduced.^26^, ^27^, ^28^ Extensive evidence links angiotensin II to profibrotic effects in lung parenchyma.^18^^,^^29^, ^30^, ^31^ Angiotensin II promotes fibrosis through multiple pathways, including myofibroblast formation, increased fibroblast motility, growth factor synthesis (eg, transforming growth factor-β), and the induction of epithelial cell senescence.^26^^,^^28^^,^^31^^,^^32^

ACE inhibitors have shown their ability, both in vivo and in vitro, to inhibit fibroproliferative processes and limit extracellular matrix deposition. By inhibiting ACE, ACE inhibitors reduce angiotensin II production,^33^ thereby halting downstream profibrotic effects such as production of transforming growth factor-β.^34^ Notably, results of animal models suggest that pirfenidone, an established IPF treatment, may exert its antifibrotic effects partly through the renin-angiotensin system.^35^ Outside of effects on the lungs, ACE inhibitors have shown efficacy in inhibiting cardiac fibrosis and preventing ventricular remodeling through inhibition of angiotensin II.^15^^,^^16^ Given the high prevalence of cardiovascular comorbidities in patients with IPF, the cardioprotective effects of ACE inhibitors may also contribute to improved outcomes, either directly or through complex interactions between cardiac and pulmonary pathologies.

A key strength of the current analysis is the real-world nature of the data, drawn from electronic health records of a large unrestricted patient population, making the findings generalizable to broader IPF populations. The robustness of these findings and their clinical utility underscore the need for future prospective trials to evaluate the efficacy of ACE inhibitor therapy in IPF, either as monotherapy or in combination with antifibrotic agents, given the established safety profile of ACE inhibitors.

There are limitations to the current analysis. The retrospective observational design restricts our ability to infer causation. In addition, despite propensity score matching, potential residual confounding factors may remain, including unmeasured effects from comorbidities and other prescribed medications. The diagnosis of IPF relied on electronic health record codes, which may introduce inaccuracy due to possible miscoding; however, survival rates in the study cohort align with expected IPF life expectancy, suggesting limited miscoding. Lastly, we were unable to assess IPF disease severity (eg, lung function or imaging data) or hospital admissions, nor did we have access to antifibrotic treatment data, as the current study period spanned different IPF diagnostic guidelines and updates.

## Interpretation

This study found that ACE inhibitor use is independently associated with reduced all-cause mortality in a large, real-world population of patients with IPF. These findings highlight the value of investigating well-established medications for new therapeutic roles in progressive diseases such as IPF, in which current treatment options are limited. We encourage the design and execution of prospective trials to validate these findings and explore ACE inhibitors as a therapeutic option in IPF.

## Funding Support

This research was funded in whole or in part by the Wellcome Trust [209553/Z/17/Z]. J. J. was supported by a 10.13039/100010269Wellcome Trust Clinical Research Career Development Fellowship 209553/Z/17/Z, Wellcome Trust Career Development Fellowship 227835/Z/23/Z, and the NIHR Biomedical Research Centre at University College London. B. O. holds a PhD scholarship sponsored by the Turkish Ministry of National Education. R. C. is supported by a 10.13039/501100000272National Institute for Health and Care Research academic clinical fellowship.

## Financial Disclosures

The authors have reported to *CHEST* the following: J. J. declares fees from Boehringer Ingelheim, F. Hoffmann–La Roche, GlaxoSmithKline, NHSX, Takeda, Wellcome Trust, Gilead Sciences, and Microsoft Research unrelated to the submitted work; and UK patent application numbers 2113765.8 and GB2211487.0. W. W. received scientific grants from Roche, Boehringer Ingelheim, Galapagos, and Alentis. None declared (B. O., R. C., T. F., M. V., M. Q. U. A., N. F., H. H., K. D.).

## Supplementary Data

e-Online Data
