# Supplementary material for: Mortality Outcomes and Angiotensin-Converting Enzyme Inhibitor Use in Patients With Idiopathic Pulmonary Fibrosis
Source: Chest. 2025 Aug 14;169(1):139–47. doi: 10.1016/j.chest.2025.07.4077 (PMC12895332; doi:10.1016/j.chest.2025.07.4077)
Supplement: e-Online Data [file mmc1.docx]

# Supplementary Materials

## Tables:

| Variable | HR (95% CI) | p-value |
| --- | --- | --- |
| ACEI | 0.99 (0.92 - 1.06) | 0.715 |
| Heart Failure | 1.23 (1.13 - 1.33) | <0.001 |
| Hypertension | 1.09 (1.01 – 1.17) | 0.027 |
| Chronic Kidney Disease | 1.16 (1.06 - 1.26) | 0.0006 |
| Pulmonary Hypertension | 1.33 (1.14 - 1.56) | 0.0003 |
| Diabetes | 1.17 (1.08 - 1.27) | 0.0002 |

**Supplementary Table 1:** Univariable Cox proportional hazards regression results for individual predictors of all-cause mortality in patients with idiopathic pulmonary fibrosis (IPF). Hazard ratios (HRs) represent the risk associated with each predictor in isolation, along with corresponding 95% confidence intervals (95% CI) and p-values.

## Figures:


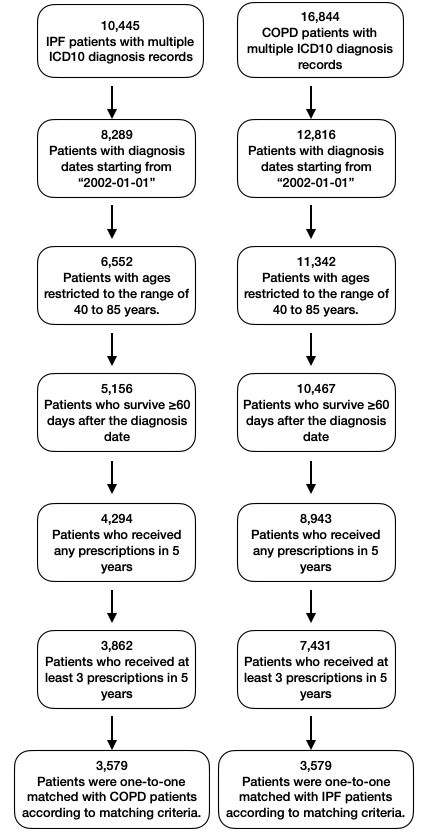


**Supplementary Figure 1:** This diagram illustrates the inclusion and exclusion criteria applied during the formation of the study cohorts for patients diagnosed with idiopathic pulmonary fibrosis (IPF) and chronic obstructive pulmonary disease (COPD). It details the number of patients excluded at each stage and provides the final sample size for analysis, stratified by ACE inhibitor use.


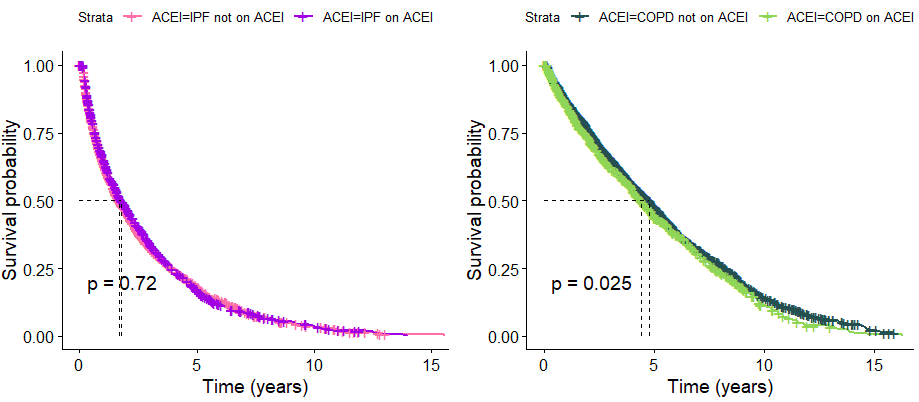


**Supplementary Figure 2:** Kaplan-Meier survival curves illustrating unadjusted survival probabilities for patients with idiopathic pulmonary fibrosis (IPF), stratified by angiotensin-converting enzyme inhibitor (ACEi) use (users vs. non-users). Median survival times are indicated by dashed lines. The p-value derived from the log-rank test indicates no significant survival difference between ACEi users and non-users (p = 0.72).


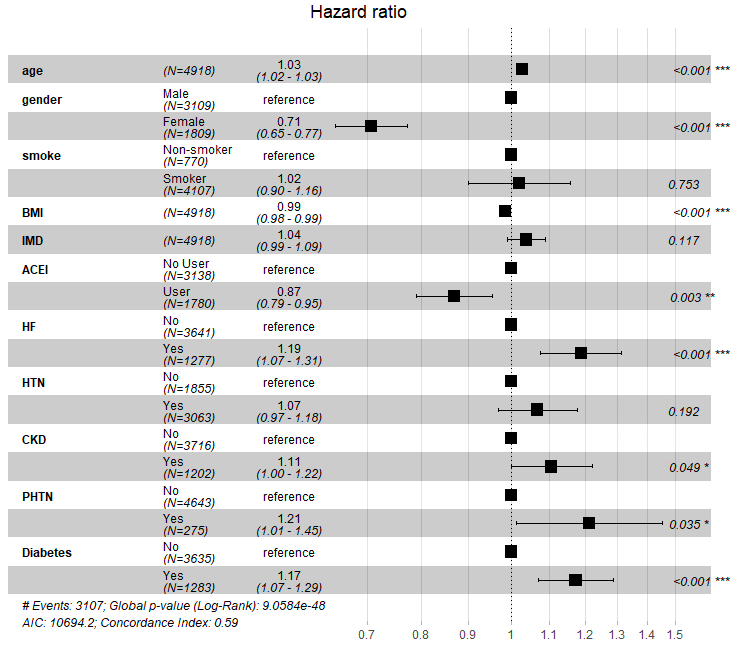


**Supplementary Figure 3:** Sensitivity Analysis of ACEi Use and Mortality in IPF Patients (without 60-day exclusion criterion). Forest plot displaying results from a multivariable Cox proportional hazards model assessing the association between angiotensin-converting enzyme inhibitor (ACEi) use and all-cause mortality in idiopathic pulmonary fibrosis (IPF) patients, without applying the 60-day exclusion restriction. Hazard ratios (HRs) are adjusted for age, gender, smoking status, body mass index (BMI), indices of multiple deprivation (IMD), heart failure (HF), hypertension (HTN), chronic kidney disease (CKD), pulmonary hypertension (PHTN), and diabetes mellitus. HRs are presented with corresponding 95% confidence intervals and p-values.
